# Online Detection of Driver Fatigue Using Steering Wheel Angles for Real Driving Conditions

**DOI:** 10.3390/s17030495

**Published:** 2017-03-02

**Authors:** Zuojin Li, Shengbo Eben Li, Renjie Li, Bo Cheng, Jinliang Shi

**Affiliations:** 1 College of Electrical and Information Engineering, Chongqing University of Science and Technology, Chongqing 401331, China; cqustlzj@sina.cn; 2 State Key Lab of Automotive Safety and Energy, Department of Automotive Engineering, Tsinghua University, Beijing 100084, China; lisb04@gmail.com (S.E.L.); lirenaxe@gmail.com (R.L.)

**Keywords:** steering wheel angles (SWA), approximate entropy (*ApEn*), warping distance, fatigue detection

## Abstract

This paper presents a drowsiness on-line detection system for monitoring driver fatigue level under real driving conditions, based on the data of steering wheel angles (SWA) collected from sensors mounted on the steering lever. The proposed system firstly extracts approximate entropy (ApEn) features from fixed sliding windows on real-time steering wheel angles time series. After that, this system linearizes the ApEn features series through an adaptive piecewise linear fitting using a given deviation. Then, the detection system calculates the warping distance between the linear features series of the sample data. Finally, this system uses the warping distance to determine the drowsiness state of the driver according to a designed binary decision classifier. The experimental data were collected from 14.68 h driving under real road conditions, including two fatigue levels: “wake” and “drowsy”. The results show that the proposed system is capable of working online with an average 78.01% accuracy, 29.35% false detections of the “awake” state, and 15.15% false detections of the “drowsy” state. The results also confirm that the proposed method based on SWA signal is valuable for applications in preventing traffic accidents caused by driver fatigue.

## 1. Introduction

Drowsiness seriously impairs peoples’ ability to drive, as they find it difficult to maintain their attention on the task. This is a harmful risk on the road. It is reported that 35%–45% of road accidents are caused by drowsy driving (i.e., driving while sleepy or fatigued) [1,2], which is why more and more studies are motivated to design an automatic detector to deal with this dangerous problem.

Recent studies have focused on two methods—intrusive and non-intrusive—to detect driver drowsiness. Intrusive detection analyzes the psychological state of the driver through electroencephalographic (EEG) and electrooculographic (EOG) information features [3,4,5,6,7,8]. Generally, the fatigue detection systems based on EEG and EOG signals provide high accuracy; however, they rely on physiological information measured by sensors located on or around the driver, and the driver’s movement therefore affects the reliability of data collection. Non-intrusive methods provide a fatigue warning based on the facial features [9,10] or the behavioral characteristics [11,12,13,14,15] of the driver. Facial features vary under different lighting conditions in the video frame, which affects the performance of the fatigue detection system. Behavioral characteristics, including steering wheel movement (SWM), standard deviation of the lane position (SDLP), and steering wheel angle (SWA) are excellent because they are reliable, real-time, and non-invasive, as the sensors embedded in various places inside the vehicle can acquire the operating information accurately and in real time. These characteristics have already demonstrated great importance in driving assistance systems [16,17,18].

The SWM method analyzes the steering wheel movement data collected from sensors mounted on the steering lever [19,20,21]. It measures the fatigue state based on the frequency of minor steering corrections [22]. When the driver is in a drowsy state, the frequency of his steering corrections reduces markedly. To avoid the interference of lane-changing, researchers usually conduct this test when only small steering angles are required. The SWM method is very reliant on the geometrical features of the road. The method can only work in certain situations.

SDLP evaluates the driver’s drowsiness level through an external camera, which tracks the position of the lane [23,24,25]. An experiment conducted by Ingre et al. [23] derived several statistical features based on SDLP, and found that when Karolinska Sleepiness Scale (KSS) ratings increase, SDLP (meters) also increases. However, it does not show consistent results on all subjects. For some subjects, the KSS ratings are very high, while SDLPs do not increase accordingly. Therefore, there are two shortcomings for this method: first, its robustness is not satisfactory; second, it is highly affected by external factors, such as lane marks, temperature, lighting, etc. In addition, drivers under the influence of alcohol and drugs will show the same SDLP features.

These reported systems have achieved good results [15]; however, the recorded data are analyzed in a simulated environment. Few reports have focused on fatigue detection under real driving conditions; this complexity will bring non-linearity, time–space variation, and instability to the driving event. This paper proposes an online fatigue detection method based on SWA data collected from sensors mounted on the steering lever under real driving conditions. The core of our method is to extract useful information from SWA data and work online. Firstly, it extracts an approximate entropy from the SWA time series within fixed time windows. Next, it gives linear expressions to the approximate entropy (ApEn) feature series within the given deviation. After that, it calculates the warping distance between the linear features on-line. Finally, the system determines drivers’ fatigue states: “awake” or “drowsy” by measuring the calculated warping distance in real time.

The remainder of this paper is structured as follows: Section 2 reviews the available ApEn of time series with a nonlinear dynamic parameter in order to discover their irregularity. There is also a brief introduction to the proposed SWA-based online drowsiness detection system, including extracting features from the SWA data, making piecewise self-adaptive linear expressions for the non-linear ApEn, calculating the warping distance between linear features for measuring its similarity to fatigue states, and designing a fatigue level classification method. Section 3 shows the experimental results for the online detection of driver fatigue based on SWA data collected from sensors situated in cars running on real roads. Section 4 discusses the performance of the proposed method under real driving conditions. Then, the conclusion is given in Section 5.

## 2. Methodology

**Figure 1:**
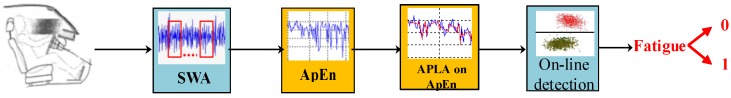
Steering wheel angle (SWA)-based on-line fatigue detection methodology. *ApEn*: approximate entropy; APLA: adaptive piecewise linear approximation.

The SWA-based fatigue detection method firstly extracts the approximate entropy from SWA time series within fixed time windows. Then, the ApEn series are given piecewise self-adaptive linear expressions. Following that, the warping distance between linear feature series is used to measure its similarity to fatigue states, so as to activate the online fatigue level detection. Finally, the driver fatigue level detection system performance is decided on-line by a designed binary classifier, as shown in Figure 1.

### 2.1. Extracting Approximate Entropy from Steering Wheel Angle

In previous studies [26,27,28,29], abnormal features in steering wheel operating behaviours appeared more frequently under drowsy situation than under waking situation. The SWA data we use for analysing and recognizing drivers’ fatigue levels is usually hidden and nonlinearly distributed. However, the hidden nonlinear features extracted under different drowsy situations are very important signs in driver fatigue level detection.

The irregularity analysis of evaluating the nonlinearity of dynamical signals is considered to be an effective approach. Since (1) changes in the physiological and behavioural process of the driver can be characterized by ApEn [30,31] and (2) irregularity and complexity of the ApEn can be quantified through ApEn [32,33], the irregularity of the stochastic time series of the steering wheel angle is therefore able to be quantified by ApEn features. In addition, the ApEn has recently been used popularly in some potential applications to quantify irregularity and complexity in stochastic time series [32,33].

We can obtain a robust estimate of ApEn from short noisy time series data. Let SWA time series data be an input; the ApEn is calculated as:

(1) $$\begin{array}{ccc} ApEn_{SWA}\left(m,r,N\right) & = & \frac{1}{N-m+1}\sum_{i=1}^{N-m+1}log\left(C_{i}^{m}\left(r\right)\right)\\ & & -\frac{1}{N-m}\sum_{i=1}^{N-m}log\left(C_{i}^{m+1}\left(r\right)\right)\\ C_{i}^{m}\left(r\right) & = & \frac{B_{i}}{N-m+1} \end{array},$$

where *m* is the number of embedded dimensions, *r* is the scale or tolerance parameter, *N* is the total number of data points in an observation period or the length of inputted time series, and $B_{i}$ is the number of *j* such that d(X(i),X(j))≤r at *i*, in which X(·) represents the *m*-dimensional vectors reconstructed from inputted time series $X_{SWA}=\left\{X_{SWA}\left(1\right),X_{SWA}\left(2\right),\ldots ,X_{SWA}\left(N\right)\right\}$:

(2) $$\begin{array}{ccc} X(i) & = & \left[X_{SWA}\left(1\right),X_{SWA}\left(i+1\right),\ldots ,X_{SWA}\left(i+m-1\right)\right],X_{SWA}\left(i\right)\in R^{m}\\ X(j) & = & \left[X_{SWA}\left(j\right),X_{SWA}\left(j+1\right),\ldots ,X_{SWA}\left(j+m-1\right)\right],X_{SWA}\left(j\right)\in R^{m} \end{array},$$

where d(X(i),X(j)) is a measure of the distance between vectors X(i) and X(j), i,j=1,…,N−m+1. The maximum difference between corresponding elements is determined by the distance.

When calculating ApEn, the growth value of *m* is added to the engineering computing workload. Meanwhile, the instantaneous change of steering wheel control variables is also increased; this will further affect the features of drowsiness in the SWA data. According to Yentes [34] and Pincus [35], we set m=2 and r=0.2×SD in this paper. SD here is the standard deviation of a fixed 2 seconds sliding window.

### 2.2. Representing ApEn Series with Adaptive Piecewise Linear Approximation

The ApEn series demonstrates the non-linear variation features of SWA data, but they cannot describe the fatigue level directly. Linear segmentation is a classic method of expressing time series, whose application to ApEn is to segment and extract basic variation modes that are relatively independent. Moreover, the linear expressions of time series are good in morphology and segment ability, so, after processing, they can be naturally segmented into different linear segments according to their variation forms. Every segment can clearly demonstrate the variation features of the time series in this given period of time, and each segment is relatively independent morphologically. To sum up, it is believed that one linear segment can represent a relatively independent variation mode. The linear approximation method of time series is shown below:

(3) $$B_{i}=\left\{\begin{array}{c} \hat{Y}=b_{0}+b_{1}X\\ e_{i}=Y_{i}-{\hat{Y}}_{i}\\ b_{1}=\frac{\sum_{i=1}^{n}\left(X_{i}-\bar{X}\right)Y_{i}}{\sum_{i=1}^{n}{\left(X_{i}-\bar{X}\right)}^{2}}\\ b_{0}=\bar{Y}-b_{1}\bar{X} \end{array}\right.,$$

where, $\left(X_{i},Y_{i}\right)$ represents the observed value of the series, $\left(X,\hat{Y}\right)$ represents the linear approximation value of the observed value, $b_{1}$ and $b_{0}$ stand for the estimated value of the linear coefficient approximated under the principle of least squares, $e_{i}$ is the residual of the observed value to the approximated line, and the condition of adaptive piecewise linear approximation (APLA) of the observed value is $e_{i}=0.2$.

### 2.3. Calculating Warping Distance of Dynamic Time Series

Dynamic time warping (DTW) is a widely applied measurement method for time series that has given satisfactory results in data clustering, classification, pattern discovery, and similarity searching. This method can conduct a warping measurement for time series of unequal lengths, and possesses robustness for abnormal data.

DTW obtains an optimal warping route by adjusting the relationship between the correspondent elements of different points in the time series, giving a better measure of the relationship between time series. Suppose there are two time series: $Q=\left\{q_{1},q_{2},q_{3},\ldots ,q_{N_{Q}}\right\}$ and $C=\left\{c_{1},c_{2},c_{3},\ldots ,c_{N_{C}}\right\}$, where $N_{Q}$ and $N_{C}$ represent the lengths of *Q* and *C*, respectively. DTW finds the optimal warping route between the two series to obtain the minimum distance value, as shown in Equation (4):

(4) $$\begin{array}{ccc} DWT(Q,C) & = & \gamma (N_{Q},N_{C})\\ \gamma (i,j) & = & d\left(i,j\right)+min\left\{\begin{array}{c} \gamma (i,j-1)\\ \gamma (i-1,j-1)\\ \gamma (i-1,j) \end{array}\right. \end{array},$$

where $i=1,2,\ldots ,N_{Q}$, $j=1,2,\ldots ,N_{C}$, γ(0,0)=0, γ(0,∞)=γ(∞,0)=∞, and in this paper, $d\left(i,j\right)={\left(q_{i}-c_{j}\right)}^{2}$.

### 2.4. Detecting Fatigue Patterns Online

When the warping distance is obtained in Equation (4), we then set an online discriminate criterion for fatigue level detection. We mark the fatigue level of the driver with “0” and “1” based on a decision making model. These stand for the two fatigue states: “awake” and “drowsy”. The detection method of the SWA-based online driver fatigue state is shown as in Equation (5):

(5) $$F_{DETEC}=\left\{\begin{array}{cc} 0\;\;\;if\; & DWT(S,S_{j})<\lambda \\ 1 & else \end{array}\right.,$$

Here, $F_{DETEC}\in \left(0,1\right)$ is the output of the fatigue level in the binary decision model, *S* stands for the standard linear time series acquired through online unsupervised learning. The learning method is shown below:

(6) $$\left\{\begin{array}{c} S⟵S_{i}\;:\;if\;Length\left(S_{i}\right)>Length\left(S\right)\\ Length(S)=j-i,(var(S(i):S(j))<0.01) \end{array}\right.,$$

where var(x) represents the variation of the *x* series. $S_{i}$ in Equation (5) stands for the linear fitted value of the SWA time series in the *i*–th time window acquired through Equation (3). The value of *λ* in Equation (5) is determined as Equation (7):

(7) $$\lambda =\left\{\begin{array}{cc} 0.31, & if\;\;Length(S)<L-1\\ 1, & else \end{array}\right.,$$

where *L* is the length of ApEn series of the sample SWA data. The sampling period set was one minute, L=30.

## 3. Experiment and Results

### 3.1. Experiment Setup

**Figure 2:**
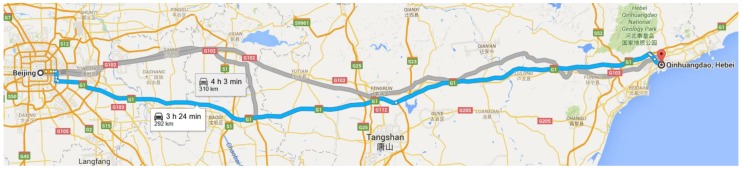
The blue line is the driving route from Beijing to Qinhuangdao, China.

Due to the monotonous driving environment on motorways, drivers easily feel tired. According to related research, 66% of accidents are caused by tired driving, 60% of accidents happened when speed was over 80 km/h, and 80% of accidents happened when speed was over 60 km/h [36]. We therefore conducted the experiment along a driving route from Beijing to Qinhuangdao, China, as shown in Figure 2. All drivers participating in this experiment were required to hold a valid driver’s license and must have had at least one year of driving experience. The experiment started after lunch when people were prone to be sleepy. The recording system named VBOX was donated by China FAW (First Automobile Works) Group Corporation. In addition, the sensors also collected data including SWA, Brakefroce, Leftsteer, Rightsteer, Can_braking, Can_thrott, YawRate, X_Accel, Y_Accel, and synchronous facial video of a driver during the driving.

The experiment was formed in two stages: firstly, divers got 15 min’s preparation to familiarize themselves with the driving condition, then they took a 90 min drive. The driving speed was set as 100 km/h for all drivers, while they were allowed to adjust the speed and position depending on the real road situations. Note that we decided on the 90 min observation time based on the ground theory that most people may get tired in monotonous driving conditions after 60 min. The 90 min of driving is therefore able to provide data for both fatigue states: “awake” and “drowsy”.

We kept quiet during the experiment, and set all cameras in the driving cabinet for recording the facial expressions of the drivers at the rate of 15 Hz. Data including SWA, brakeforce, leftsteer, rightsteer, can_braking, can_thrott, YawRate, X_Accel, Y_Accel, etc. were recorded at the rate of 100 Hz. SWA data were able to represent operating behavior characteristics of a driver more significantly [36]. Compared to other recorded signals, SWA made the greatest contribution to the driver’s fatigue state recognition, and so we restricted our focus to SWA data. The average age of the participating drivers was 39.6, and the average driving experience was 9.6 years. The total experiment time exceeded 20 h. Because four observed divers did not show drowsy state during the experiment, we only selected data from six valid drivers’ data in this paper. The total experiment time of the six drivers was over 14 h.

### 3.2. Fatigue Level Criteria

It is necessary to obtain prior knowledge of fatigue levels from sample data for both operating features extraction and detection model design and construction. In order to label the fatigue level of the corresponding data samples, we require an accurate and reliable measuring method to evaluate the real fatigue level of the driver. We therefore invited three experts to join our experiment.

There are three steps to constructing a qualified sample data of driver’s fatigue level: segmentation, evaluation, and visualization of the sample experiment data.

#### Segmentation Synchronous Facial Videos and Operating Feature Data

We clipped facial videos into 1-min segments by video clipping software, and then the operating feature data were spliced by starting time and ending time of the video clips, accordingly.

According to [36], we used video-and-expert method, which evaluates the video clips based on the facial fatigue level evaluation criteria. The three experts evaluate all facial video samples based on this criteria chart according to the time windows. The inconsistent results can be marked directly by the three experts without argument; otherwise, they will negotiate the results until an agreement is reached. If the agreement cannot be made, the sample would be discarded.

After that, we visualize the samples; if a curved line or lane shifting appears, we discard the sample. Finally, the qualified sample data with fatigue level labels are stored in the sample database. Table 1 shows the criteria for fatigue level evaluation.

### 3.3. Experiment Results

**Figure 3:**
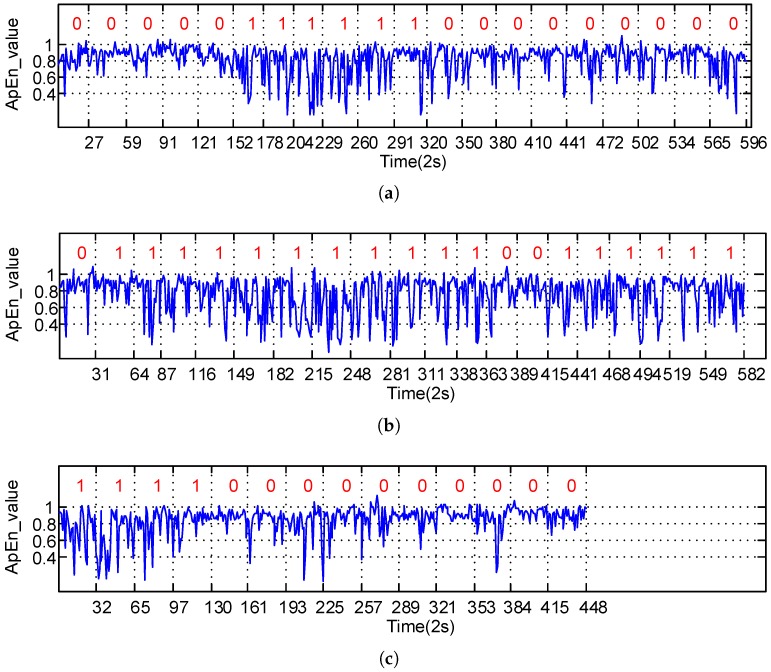
An example of ApEn distribution. Red numbers mark fatigue levels: 0 is “awake”, 1 is “drowsy”. (**a**) ApEn distribution of Subject 6’s fatigue states in the first 20 samples; (**b**) ApEn distribution of Subject 6’s fatigue states in the following 20 samples; (**c**) ApEn distribution of Subject 6’s fatigue states in the last 14 samples.

The ApEn was computed for all samples, taking Subject 6 as an example. The ApEn distribution of the SWA time series under real driving conditions is shown in Figure 3. The horizontal axis represents numbers of short time sliding window of the sample, and the vertical axis represents the computing results of ApEn for each window in each sub-graph. Red marks 0 and 1 are the label of two fatigue levels, respectively—namely, “awake” and “drowsy”—evaluated by the experts. Figure 3a shows the ApEn distribution of Subject 6’s fatigue states in the first 20 samples, Figure 3b shows the following 20 samples, and Figure 3c demonstrates the last 14 samples. As shown in these figures, the ApEn values of the SWA data is mainly distributed in the interval $\left[0.8\;\;1\right]$ when the driver is in the “awake” state, “0”, and those values are distributed more widely in the interval $\left[0.4\;\;1\right]$ when the driver is in the “drowsy” state. Thus, there is evidence that the ApEn value distribution of the driver’s SWA data represents a noticeable difference between the two fatigue levels. This allows us to input more details into the ApEn and explore the driver’s fatigue features from the SWA data and detect the fatigue level.

**Figure 4:**
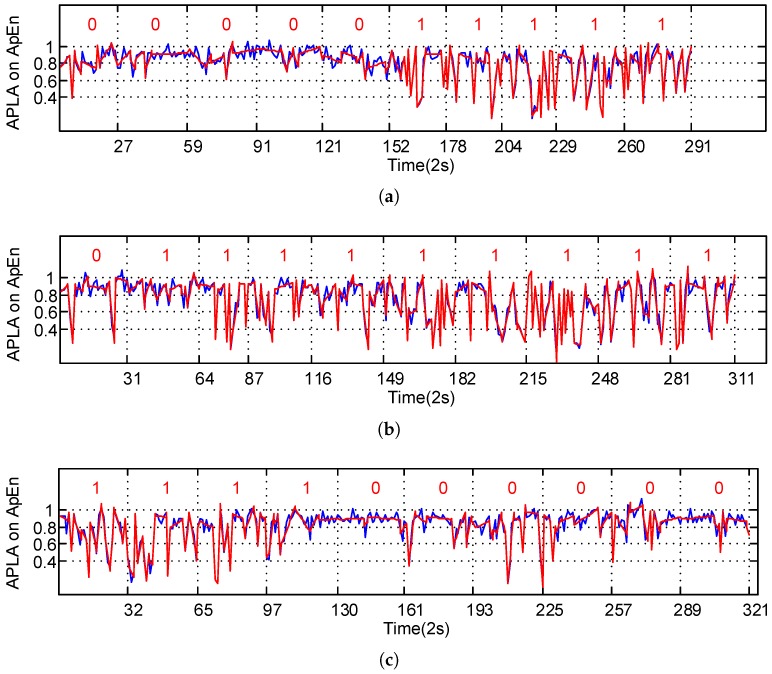
Adaptive piecewise linear approximation (APLA) of ApEn on thirty samples. Red numbers mark fatigue levels: 0 is “awake”, 1 is “drowsy”. (**a**–**c**) show the APLA distribution for ApEn of Subject 6’s fatigue states in 10 non-duplicate samples from 3 groups.

Figure 4 shows the results of adaptive piecewise linear approximation of ApEn series in Equation (3). Blue curves represent the ApEn series of the sample, and red curves represent their linear approximation results. This shows that the red lines can evidently express the changing trend of ApEn series in the correspondent segment, and thus demonstrate the inherent feature. It can be concluded that linear approximation simplifies the ApEn data distribution, which reflects their morphological features. This finding provides more visible grounds for determining the fatigue levels. Moreover, as seen from this figure, when the ApEn series distribution shows great changes, the approximated lines can be naturally segmented into different forms. Each segment—relatively independent in morphology—can directly reflect the variation features of the time series in the given period of time. Figure 4 also shows that each adaptive linear segment can represent a relatively independent variation mode and describe the inherent rules of the series.

**Figure 5:**
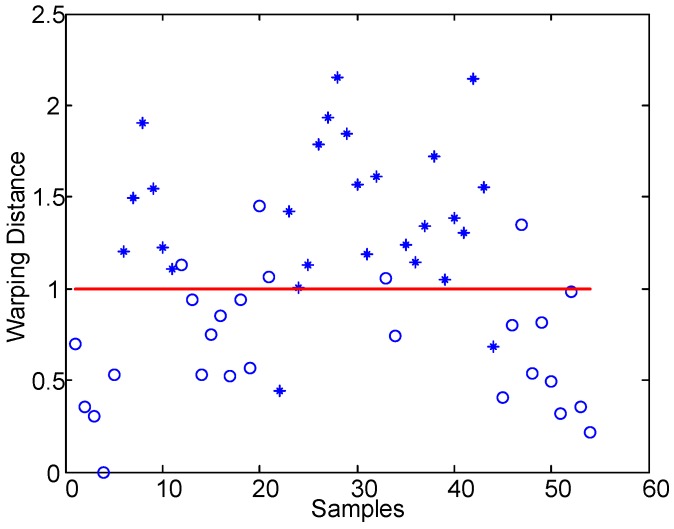
The computing result of the warping distance of subject 6.

Figure 5 shows the results of the warping time distance between series calculated in Equation (4) based on Subject 6, where “o” represents the sample that is in the awake situation and “*” represents the sample in the drowsy situation. We can see that the warping distances of the samples under different fatigue levels show distinct distribution intervals. According to Equation (6), we set the threshold to determine fatigue levels (shown with the red line), which can clearly divide drowsy and awake samples.

The rate of accuracy (AC), false positive (FP), and false negative (FN) of the data set are usually used to measure the performance of the detection system. Their calculation is shown below:

(8) $$AC_{rate}=\frac{TP+TN}{TP+FP+TN+FN}\times 100\%$$

(9) $$FP_{rate}=\frac{FP}{FP+TN}\times 100\%$$

(10) $$FN_{rate}=\frac{TN}{TP+FN}\times 100\%$$

where TP is the number of true positives, TN is the number of true negatives, FP is the number of false classification for positive samples; and FN is for negative samples. Their relationship is shown in Table 2. AC indicates the overall detection accuracy for both positive and negative patterns.

We evaluate the proposed method in a confusion matrix based on Table 2. The columns refer to experts’ decisions, and the rows represent the classification performed by the proposed method. The number of testing samples presented at each level is displayed in the last row. Usually, experiments only focus on the accuracy rate, while paying no attention to missed alarms. However, we are supposed to find a good compromise between a high accuracy rate for fatigue level and a low number of missed alarms. Indeed, a large number of false alarms may discredit the system, but a high rate of missed alarms is potentially risky for a driver.

Similarly, we applied the proposed online detecting method to all samples of the six subjects. The fatigue levels are divided into two levels: awake and drowsy, represented with the output labels “0” and “1”. The testing results are shown in Table 3. We can see that there are 191 samples in the database, of which 92 are awake and 99 are drowsy. The system achieved 78.01% accuracy for all samples, with 70.65% true negatives and 29.35% false positives. It also shows 84.85% true positives, with 15.15% false negatives.

## 4. Discussion

The proposed method uses SWA data to detect drivers’ fatigue levels. The mental state of a driver can be directly reflected onto his/her driving behavior, the most frequent and sensitive of which is steering wheel operation. This online detection method allows the detection of two fatigue levels: “awake” and “drowsy” under the real driving conditions. The detection system achieves a good performance with 78.01% of fatigue levels correctly classified in about 15 h driving on real roads. As a result, the extracted information from SWA data promotes the performance of fatigue detection with robustness and reliability as verified in real-road experiments. The experiments confirm that SWA data are closer to the real driving conditions, and can better reflect the mental state and operating behaviors of drivers. Meanwhile, the detecting system shows a tolerable rate of false alarms or false positives (29.35%) and missed alarms or false negatives (15.15%). This system is also robust because the evaluation criteria (see Table 1) decided by three experts are tested with various methods, which are based on facial video information rather than SWA information, including facial expressions, head positions and mental state.

The online method proposed in this paper extracts the real-time ApEn features from the SWA time series, then conducts a self-adaptive linear segmentation to them, and finally calculates the warping distance between the linear feature series in the present time frame and the reference time frame to determine the fatigue state. The ApEns extracted from SWA reflect the variation features of the driver’s operating modes. When the driver is tired, his/her capacity to act will be reduced, and the time frame of the SWA series transformation will become larger; in other words, fewer new patterns will emerge in the activity time series. As shown in Figure 3, when the driver is very tired, the ApEn of the SWA series is small, and the overall distribution of the ApEn shows large fluctuations. To further explore the distinctive features between the drowsy state and waking state, we use linear fitting to reflect the local changing trend of the ApEn series and the self-adaptive segmentation to represent the morphological distribution features of time series. As shown in Figure 4, red lines can reliably reflect local variation features of time series and the inherent rules of the data, and accordingly more directly demonstrate the state modes of time series. Besides, compared with fixed-length linear segmentation, the self-adaptive segmentation proposed in this paper is more suitable for the online testing of fatigue states. The latter is commonly applied in fatigue testing; however, because of the complexity of driving conditions, the data lengths within the fixed-length time frame may vary. That is why the application of Euclidean distance to measure the similarity of time series can hardly satisfy needs under real driving conditions. The dynamic warping time distance can acquire an optimal warping route by adjusting the relationship between the corresponding elements of different points of time in the time series, so it can better measure the relationship between the time series. Evidently, this method satisfactorily solves the problem brought by unequal lengths of time series. This paper adopts dynamic warping distance between series to measure their similarity. If the distance is small, it is deemed that the tested series is highly similar to the reference series; i.e., the two samples are in the same fatigue state. As seen from Figure 5, the warping distance between the “awake” sample and reference is small; that is because the reference sample always maintains the awake condition under real driving conditions, through unsupervised learning in Equation (6). Contrarily, the warping distance between the series of the drowsy sample and that of the reference is large, so the driver shows a fatigue level different from that of the reference sample. Considering that the driving habits may vary, Equation (7) proposes the judgment threshold, which is suitable for all samples in this paper. For a larger sample, this threshold may need further verification or improvement.

However, as there are not many existing studies that report on SWA fatigue detection based on real road experiments, we are not able to show our superiority in comparison with other methods. We only reflect on Qu’s work [36] in order to evaluate our results. He did fatigue level detection with SWA data in a laboratory environment, having an accuracy rate of 86.1%, higher than the 78.01% of this paper; the average rate of false positives was 12.09%, lower than the 29.35% in the current study; while the rate of false negatives in Qu’s work was 18.47%, higher than the 15.15% of our work. In fact, the SWA data retrieved from real driving conditions are extremely difficult to analyze compared to those from the laboratory environment, considering that random vibration may cause the SWA series to drift dramatically. The irregular drifting time series existing in the original data were recognized as false statistic features. Therefore, although the proposed detection method shows slightly poorer performance than that based on simulation in the laboratory, it possesses greater value in its engineering application.

## 5. Conclusions and Future Work

This paper presents an on-line drowsiness detection system based on SWA information retrieved from sensors located in fixed positions, tested with 14.68 h real road driving. The proposed system firstly extracts ApEn from fixed sliding windows on a SWA time series. Then, it linearizes the ApEn features series through adaptive piecewise linear fitting within a given deviation. Following that, the system calculates the warping distance between the linear features series of the sample data. Finally, this system determines the drowsiness state based on the warping distance. The experimental data contains two fatigue levels: “awake” and “drowsy”, according to the facial expressions, head position, and mental state of a driver, which have been examined by related experts.

The experiment retrieved an average accuracy rate of 78.01%, 29.35% false alarms (false positives), and 15.15% missed alarms (false negatives) in cases of two-class fatigue detection. As a result, this paper confidently confirms that the proposed on-line method is valuable for application in avoiding traffic accidents that are caused by driving in tired condition. Previous work [36] has shown that when SWA is combined with vehicle state information, like yaw angles, lateral positions, etc., it can produce a high detection rate in a simulation laboratory. Inspired by this, we will combine these types of information in real driving conditions to improve the detection rate of driver fatigue as our worthwhile future work. This could provide new information for the detection of driver fatigue.

## Figures and Tables

**Table 1:** Evaluation criteria for driver fatigue level.

Fatigue Level	Fatigue Point	Feature Description
Awake	1	Eyes keep open, wink in a very short time, rapid eyeball movement, concentrate on driving, keep the head in line, and very mobile facial expressions
Drowsy	2	Eyelid closure occurs, cannot keep eye open like normal, blink is getting slower, slower eyeball movement, eyesight is strained, yawning, deep breaths, sighing, swallowing, cannot always concentrate on driving. Eyelid closure occurs often, heavy eyelids or eyes semi-open or very hard to keep eyes open, eyes close for a long time, dozing, head cocked to one side, cannot drive

**Table 2:** Contingency table.

		Expert Decision	
		“Awake” (Level 0)	“Drowsy” (Level 1)
Automatic decision	“Awake” (level 0)	True Negative (TN)	False Negative (FN)
	“Drowsy” (level 1)	False Positive (FP)	True Positive (TP)

**Table 3:** Confusion matrix of detection drowsiness Levels “0” and “l”.

		Expert Decision	
		“Awake” (Level 0)	“Drowsy” (Level 1)
Automatic decision	“Awake” (level 0)	65	15
	“Drowsy” (level 1)	27	84
	Samples	92	99
